# Emphysematous gastritis with extensive portal venous gas and endoscopic mucosal necrosis: gastric preservation despite an alarming presentation

**DOI:** 10.1093/jscr/rjag942

**Published:** 2026-10-19

**Authors:** Ettore Caruso, Carmine Gabriele, Denise Gambardella, Roccantonio Pellegrino, Maria Bonaddio, Luigi Strangis, Francesca Ibba, Martina De Luca, Martina Saragò, Martina Nicoletti, Laura Rizzo, Francesca Vescio, Vittorio Tedesco, Aurora Somma, Giuseppe Bonadio, Gianlorenzo Schicchi, Angelo Aldo Schicchi, Manfredo Tedesco

**Affiliations:** Department of General Surgery, G. Paolo II Hospital, Via Senatore Arturo Perugini, 88046 (CZ), Lamezia Terme, Italy; Department of General Surgery, G. Paolo II Hospital, Via Senatore Arturo Perugini, 88046 (CZ), Lamezia Terme, Italy; Department of General Surgery, G. Paolo II Hospital, Via Senatore Arturo Perugini, 88046 (CZ), Lamezia Terme, Italy; Department of General Surgery, G. Paolo II Hospital, Via Senatore Arturo Perugini, 88046 (CZ), Lamezia Terme, Italy; Department of General Surgery, G. Paolo II Hospital, Via Senatore Arturo Perugini, 88046 (CZ), Lamezia Terme, Italy; Department of General Surgery, G. Paolo II Hospital, Via Senatore Arturo Perugini, 88046 (CZ), Lamezia Terme, Italy; Department of General Surgery, G. Paolo II Hospital, Via Senatore Arturo Perugini, 88046 (CZ), Lamezia Terme, Italy; Department of General Surgery, G. Paolo II Hospital, Via Senatore Arturo Perugini, 88046 (CZ), Lamezia Terme, Italy; Department of General Surgery, G. Paolo II Hospital, Via Senatore Arturo Perugini, 88046 (CZ), Lamezia Terme, Italy; Department of General Surgery, G. Paolo II Hospital, Via Senatore Arturo Perugini, 88046 (CZ), Lamezia Terme, Italy; Department of General Surgery, G. Paolo II Hospital, Via Senatore Arturo Perugini, 88046 (CZ), Lamezia Terme, Italy; Department of General Surgery, G. Paolo II Hospital, Via Senatore Arturo Perugini, 88046 (CZ), Lamezia Terme, Italy; Department of Medical and Surgical Sciences, University of Plovdiv, 15A Vasil Aprilov Boulevard, 4002 Plovdiv, Bulgaria; Department of Medical and Surgical Sciences, University of Plovdiv, 15A Vasil Aprilov Boulevard, 4002 Plovdiv, Bulgaria; Department of Anesthesia, G. Paolo II Hospital, Lamezia Terme, Via Senatore Arturo Perugini, 88046 (CZ), Italy; Department of Medical and Surgical Sciences, “Magna Graecia” University of Catanzaro, Viale Europa, Località Germaneto, 88100 (CZ), Catanzaro, Italy; Digestive Endoscopy Unit, Department of Gastroenterology, G. Paolo II Hospital, Via Senatore Arturo Perugini, 88046 (CZ), Lamezia Terme, Italy; Department of General Surgery, G. Paolo II Hospital, Via Senatore Arturo Perugini, 88046 (CZ), Lamezia Terme, Italy

**Keywords:** emphysematous gastritis, portal venous gas, gastric pneumatosis, gastric mucosal necrosis, upper gastrointestinal endoscopy, diagnostic laparoscopy, conservative management

## Abstract

Emphysematous gastritis (EG) is a rare, potentially life-threatening condition. Extensive gastric pneumatosis and portal venous gas may suggest irreversible ischemia, although radiological severity and mucosal injury do not necessarily indicate transmural necrosis. A 70-year-old man presented with acute epigastric pain and vomiting. Computed tomography (CT) demonstrated extensive gastric and duodenal pneumatosis with portomesenteric venous gas but patent visceral arteries. Immediately after CT, he developed cardiac arrest, with return of spontaneous circulation after approximately 14 min. Emergency laparoscopy showed a viable stomach without perforation or transmural necrosis, whereas intraoperative endoscopy demonstrated extensive necrotic-appearing gastric mucosa. Gastrectomy was therefore avoided. Follow-up CT 4 days later showed complete resolution of pneumatosis and portomesenteric venous gas. The patient subsequently died from post-anoxic neurological injury and multiorgan failure. Surgical exploration and gastric resection should remain distinct decisions in severe EG, integrating imaging, endoscopy, and direct assessment of gastric viability.

## Introduction

Emphysematous gastritis (EG) is a rare, potentially life-threatening condition characterized by intramural gastric gas, classically associated with gas-forming microorganisms [1–4]. It should be distinguished from gastric emphysema, a generally more benign non-infectious condition [2, 5]. Although extensive gastric pneumatosis and portal venous gas may suggest irreversible ischemia, contemporary evidence supports selective non-resective management when perforation or transmural necrosis is absent [1, 6, 7]. We report severe EG with endoscopic mucosal necrosis but preserved transmural gastric viability, successfully managed without gastrectomy.

## Case presentation

A 70-year-old man with ischemic heart disease, hypertension, dyslipidemia, and type 2 diabetes presented on 24 July 2026, with acute epigastric pain, asthenia, and bilious vomiting. He was initially hemodynamically stable (blood pressure 150/80 mmHg, heart rate 73 beats/min, SpO₂ 95%, temperature 36°C), with abdominal distension and epigastric tenderness but no peritonitis.

Urgent non-contrast computed tomography (CT) demonstrated marked gastric distension and extensive gas within the gastric venous drainage, superior mesenteric vein, and intra- and extrahepatic portal circulation (Fig. 1A). Contrast-enhanced CT demonstrated extensive pneumatosis predominantly involving the gastric fundus and body, associated with duodenal pneumatosis and portomesenteric venous gas (Fig. 1B). The celiac trunk and mesenteric arteries were patent, and no free intraperitoneal air was present. Although microbiological confirmation was unavailable, the radiological findings, subsequent systemic deterioration, and later endoscopic evidence of necrotic gastric mucosa were considered most consistent with EG. The main clinical events, diagnostic investigations, and subsequent disease progression are summarized in Table 1.

**Table 1:** Clinical timeline.

Time	Clinical event
24 July 2026	Acute epigastric pain and bilious vomiting; hemodynamically stable, no peritonitis
Initial CT	Gastric distension, gastric/duodenal pneumatosis, and extensive portomesenteric venous gas
Immediately after CT	Cardiac arrest; ROSC after ~14 min
July 25	Emergency exploratory laparoscopy + intraoperative EGD
Laparoscopy	Viable gastric wall; no perforation or transmural necrosis
EGD	Necrotic-appearing fundal and posterior body mucosa
Operative decision	Gastric preservation; no gastrectomy
July 29	Complete radiological resolution of pneumatosis and portomesenteric venous gas
Subsequent ICU course	Post-anoxic coma, KDIGO 3 AKI, thrombocytopenia, and multiorgan dysfunction
>20 days	Death from multiorgan failure

**Figure 1:**
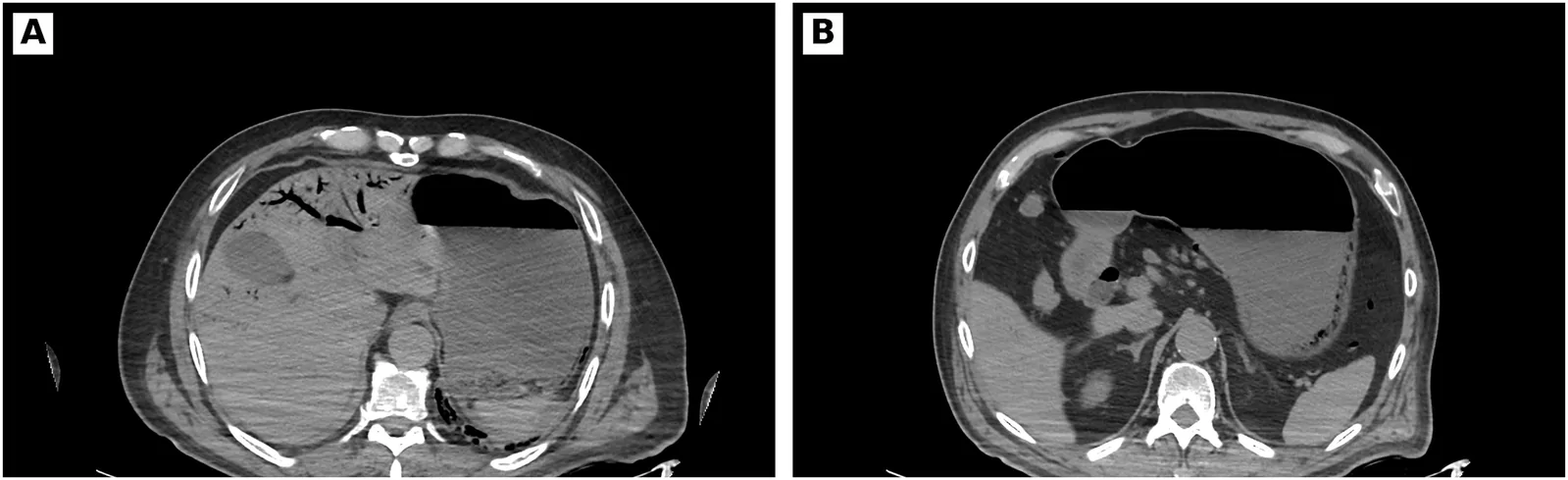
Preoperative CT findings. (A) Axial CT showing extensive branching peripheral intrahepatic portal venous gas. (B) Marked gastric distension with gastric pneumatosis and extensive portomesenteric venous gas.

Immediately after CT, the patient developed cardiac arrest, initially with pulseless electrical activity and subsequently a shockable rhythm requiring defibrillation. Return of spontaneous circulation was achieved after approximately 14 min of cardiopulmonary resuscitation. A direct causal relationship between EG and cardiac arrest could not be established.

Given the profound deterioration associated with extensive pneumatosis and portomesenteric venous gas, emergency exploratory laparoscopy was performed. A small amount of clear peritoneal fluid was found, but there was no gastrointestinal perforation or macroscopic intestinal ischemia. Exploration of the lesser sac showed a macroscopically viable stomach without full-thickness ischemia or necrosis (Fig. 2A).

**Figure 2:**
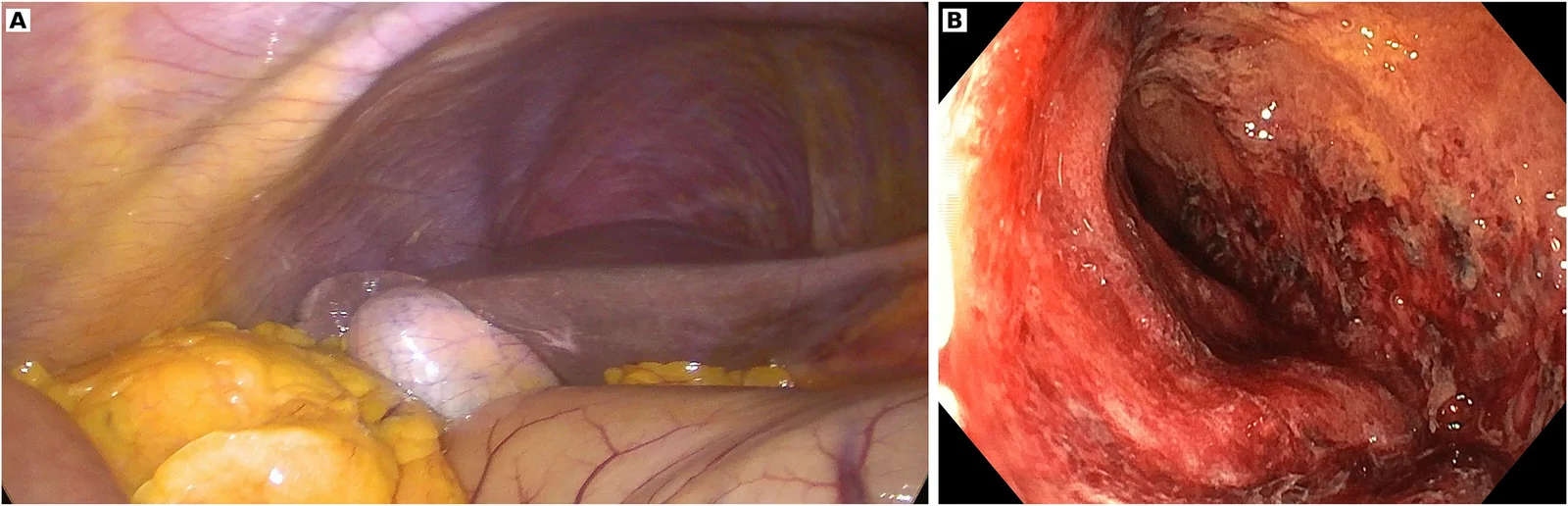
Discordance between laparoscopic and endoscopic findings. (A) Exploratory laparoscopy showing a macroscopically viable gastric serosal surface without perforation or full-thickness necrosis. (B) Intraoperative esophagogastroduodenoscopy showing extensive dark, hemorrhagic, necrotic-appearing gastric mucosa. The absence of transmural necrosis supported gastric preservation.

Intraoperative esophagogastroduodenoscopy was therefore performed. After removal of retained reddish gastric fluid, the fundus and posterior body showed extensive dark, necrotic-appearing mucosal injury, progressively returning to normal toward the prepyloric antrum; the examined duodenum appeared viable (Fig. 2B). Thus, severe mucosal injury contrasted sharply with preserved serosal and transmural gastric viability.

In the absence of perforation or macroscopic transmural necrosis, and considering the extreme physiological risk following prolonged resuscitation, gastrectomy was not performed. A peritoneal drain was placed, and the patient was transferred to intensive care, where treatment included gastric decompression, proton-pump inhibition, broad-spectrum intravenous antibiotics, including meropenem, and organ support.

Follow-up CT on July 29, 4 days after exploration, demonstrated complete resolution of gastric and duodenal pneumatosis and disappearance of the previously extensive portomesenteric venous gas, without perforation or progressive gastric necrosis (Fig. 3).

**Figure 3:**
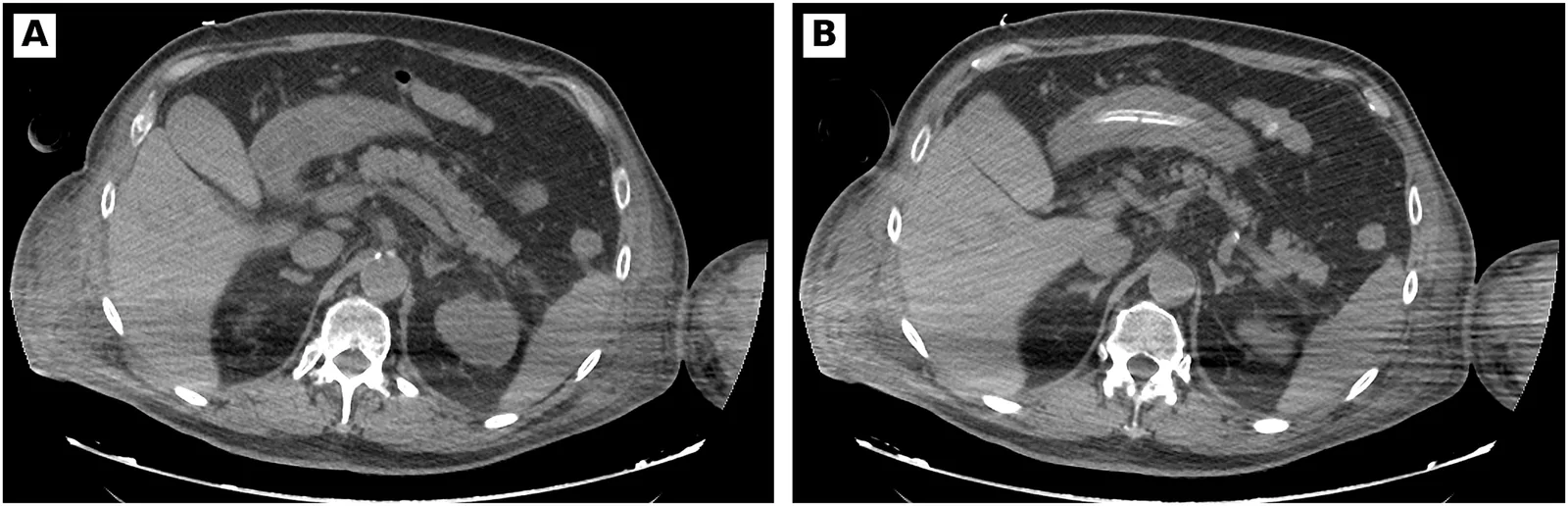
Follow-up CT 4 days after surgical exploration. Axial CT images demonstrating resolution of the previously extensive portal and portomesenteric venous gas and associated gastric abnormalities, without evidence of perforation or progressive gastric necrosis.

Despite resolution of the gastrointestinal process, the patient remained in severe post-anoxic coma. Renal function deteriorated from a serum creatinine of 0.83 mg/dl on July 25 to 7.60 mg/dl by July 31, consistent with KDIGO stage 3 acute kidney injury, accompanied by thrombocytopenia and progressive multiorgan dysfunction. He died more than 20 days after presentation. No subsequent evidence of gastric perforation or transmural necrosis was documented.

## Discussion

This case highlights a clinically important distinction in severe EG: the indication for surgical exploration and the indication for gastric resection are not synonymous. Extensive gastric pneumatosis, portal venous gas, and even necrotic-appearing mucosa may occur without irreversible full-thickness gastric necrosis.

Historically, EG carried mortality rates exceeding 50% and frequently prompted aggressive surgical treatment [2, 8]. More recent evidence indicates a shift toward conservative or organ-preserving management. In a recent systematic review of 189 adult cases, Adam *et al*. reported an overall mortality of 26.9%; antibiotics were administered in 76% of patients, whereas only 28% underwent surgery [1]. Another systematic review of 116 cases similarly found that medical management predominated [9].

Portal venous gas should not itself be considered evidence of transmural necrosis. In a multi-institutional study of 58 patients with gastric pneumatosis, Epin *et al*. identified portal venous gas in 90%, whereas bowel ischemia was present in only 29% [7]. Hemodynamic instability, guarding, arterial lactate >2 mmol/l, and absence of gastric dilatation were associated with mortality; portal venous gas alone did not mandate resection [7].

The most distinctive feature of our case was the discordance between endoscopic and laparoscopic findings. Endoscopy documented extensive necrotic-appearing mucosa, whereas direct surgical inspection demonstrated a viable gastric serosa without perforation or full-thickness necrosis. Similar dissociation has been reported by Bak *et al*., in whose patient mucosal ischemia coexisted with a viable serosal surface and gastrectomy was avoided [10]. Patel *et al*. likewise described extensive fundal ulceration and necrotic mucosa successfully managed without gastric resection [11]. These observations support the complementary roles of endoscopy, which defines mucosal injury, and surgical exploration, which directly assesses transmural viability.

The complete radiological resolution 4 days after exploration provides objective retrospective support for gastric preservation in this patient. Comparable rapid improvement has been described after non-resective management [6, 12]. Importantly, the subsequent death should not be interpreted as failure of gastric preservation: the gastrointestinal abnormalities resolved completely, while the later course was dominated by post-anoxic neurological injury, acute kidney injury, and multiorgan failure.

The absence of microbiological confirmation is an important limitation, and the available evidence cannot establish a causal relationship between EG and cardiac arrest. Nevertheless, the temporal sequence, multimodal findings, and documented radiological resolution make this case clinically instructive.

## Conclusion

Severe radiological findings in EG should prompt urgent assessment but not reflex gastrectomy. When exploration demonstrates a viable gastric wall without perforation or transmural necrosis, gastric preservation may be appropriate despite portal venous gas and severe endoscopic mucosal injury. Multimodal assessment integrating contrast-enhanced CT, endoscopy, and direct surgical evaluation can distinguish the need for exploration from the need for resection.

## Data Availability

The datasets generated and analyzed during the current study are not publicly available due to privacy considerations but are available from the corresponding author upon reasonable request.
